# Modelling experimentally measured of ciprofloxacin antibiotic diffusion in *Pseudomonas aeruginosa* biofilm formed in artificial sputum medium

**DOI:** 10.1371/journal.pone.0243003

**Published:** 2020-12-03

**Authors:** Tadeusz Kosztołowicz, Ralf Metzler, Sławomir Wa̡sik, Michał Arabski

**Affiliations:** 1 Institute of Physics, Jan Kochanowski University, Kielce, Poland; 2 Institute for Physics and Astronomy, University of Potsdam, Potsdam-Golm, Germany; 3 Institute of Biology, Jan Kochanowski University, Kielce, Poland; Universidade Estadual de Maringa, BRAZIL

## Abstract

We study the experimentally measured ciprofloxacin antibiotic diffusion through a gel-like artificial sputum medium (ASM) mimicking physiological conditions typical for a cystic fibrosis layer, in which regions occupied by *Pseudomonas aeruginosa* bacteria are present. To quantify the antibiotic diffusion dynamics we employ a phenomenological model using a subdiffusion-absorption equation with a fractional time derivative. This effective equation describes molecular diffusion in a medium structured akin Thompson’s plumpudding model; here the ‘pudding’ background represents the ASM and the ‘plums’ represent the bacterial biofilm. The pudding is a subdiffusion barrier for antibiotic molecules that can affect bacteria found in plums. For the experimental study we use an interferometric method to determine the time evolution of the amount of antibiotic that has diffused through the biofilm. The theoretical model shows that this function is qualitatively different depending on whether or not absorption of the antibiotic in the biofilm occurs. We show that the process can be divided into three successive stages: (1) only antibiotic subdiffusion with constant biofilm parameters, (2) subdiffusion and absorption of antibiotic molecules with variable biofilm transport parameters, (3) subdiffusion and absorption in the medium but the biofilm parameters are constant again. Stage 2 is interpreted as the appearance of an intensive defence build–up of bacteria against the action of the antibiotic, and in the stage 3 it is likely that the bacteria have been inactivated. Times at which stages change are determined from the experimentally obtained temporal evolution of the amount of antibiotic that has diffused through the ASM with bacteria. Our analysis shows good agreement between experimental and theoretical results and is consistent with the biologically expected biofilm response. We show that an experimental method to study the temporal evolution of the amount of a substance that has diffused through a biofilm is useful in studying the processes occurring in a biofilm. We also show that the complicated biological process of antibiotic diffusion in a biofilm can be described by a fractional subdiffusion-absorption equation with subdiffusion and absorption parameters that change over time.

## Introduction

Biological processes are very complex, so mathematical modelling of these processes is a quite difficult task. On the one hand, mathematical models are still being developed, even for relatively simple geometries, for instance, for molecular chemical reactions involved in gene regulation in bacteria cells [1–4]. On the other hand, in order to describe all important factors affecting a biological process, sometimes a dozen or even several dozen variables describing various factors and several equations governing the time evolution of these variables are used. An example of this is the modelling of the development and transport of cancer cells [5]. In practice, a system of many equations can be solved using numerical methods. The usability of a model is usually checked by comparing theoretical results with empirical data. However, a large number of fit parameters is often used when comparing theoretical and experimental results. If there are not many empirical results determined with relatively small measurement errors, the verification of such models may not be effective.

The process of transporting an antibiotic into a bacterial biofilm is such a complicated process. A biofilm has a complex structure that changes over time. Special biofilm defence mechanisms, not fully understood, that affect the diffusion of antibiotic molecules in the biofilm can be activated [6–8]. Modelling the diffusion of antibiotics in a biofilm, we therefore propose to use a different strategy. We assume that the process of diffusion through a biofilm is described by a small number of equations that can be solved analytically. These equations contain a small number of parameters that have a simple physical interpretation. These parameters are, however, allowed to change over time. The idea of this approach is that the complexity of the biological process can be manifested in complex forms of the temporal evolutions of parameters. When the biofilm parameters cease to change despite the continuous diffusion of the antibiotic through the biofilm, it can be concluded that the bacteria have stopped responding to the action of the antibiotic. An important information for biologists is at what antibiotic concentration and after what time this process will start. It is very likely that the bacteria are killed then. However, linking changes in physical parameters with biological processes is typically a challenge. In particular, we find that the time evolution of the transport parameters can be captured by simple functions with few parameters.

Nowadays, a variety of laboratory techniques are used to evaluate antibacterial properties of drugs, mainly, disk-diffusion methods as a standard in microbiological laboratories, antimicrobial gradient methods (E-tests) which allow to determine the minimum inhibitory concentration (MIC) of chemical agents, of ATP bioluminescence assay based on the measurement of adenosine triphosphate (ATP) produced by bacteria and antibiofilm screening assays [9]. The antibiofilm screening assays might be classified in three models of in vitro study: static, genetic and flow. Static assays allow to measure early stages of biofilm formation. They are based on colorymetric measurements of bacterial cells and biofilm matrix stained by crystal violet or safranin and fluorometric analysis using resazurin, SYTO-9 or propidium iodide to evaluate the metabolic activity of cells formed biofilm. Genetic techniques are used to measure the gene expression of coded proteins specific for forming biofilm, as Real Time Quantitative-Reverse Transcription-PCR (qRT-PCR). Fluid dynamics is an important factor known to influence biofilm formation in natural environments [10]. There are several methods as FC270 flow-cell system or Microfluidics belonging to the employed ‘flow’ model. In our previous studies we used a new optimized laser interferomery method to measure the biofilm degradation [11–13]. In this paper we show that the combination of this experimental method with the results provided by our theoretical model opens up new research avenues as well as offers concrete ways to interpret the observed antibiotic concentration dynamics.

Ciprofloxacin antibiotic is one of the most commonly used fluoroquinolone drugs in the treatment of *P. aeruginosa* infections during cystic fibrosis. We present a model of the antibiotic diffusion in a biofilm that can be described as a ‘plumpudding’. Antibiotic particles can interact with bacteria located in ‘plums’, pocked in the ‘pudding’ background while the pudding is a diffusion barrier for the antibiotic. In the experimental system the ‘pudding’ represents the artificial sputum medium (ASM) which mimics cystic fibrosis patient sputum. The bacteria plums correspond to bacterial cells making up microcolonies, i.e. biofilms, such as *Pseudomonas aeruginosa*. In gel–like media subdiffusion is likely to occur [14–20]. Since the ASM matrix (the pudding representing the cystic fibrosis EPS) has a gel–like consistency and has physicochemical interactions with the diffusing antibiotic, subdiffusion is therefore expected in this medium. As a result of the activation of bacterial defence mechanisms, an antibiotic particle can be permanently or temporarily retained in the plums or can be destroyed, depending on the type of bacterial defence mechanism. The process that a molecule can be permanently trapped or destroyed is effectively described by the subdiffusion-absorption equation. The process of temporary retention of a molecule can be regarded as a reversible reaction. In this case, the process is described by the subdiffusion equation in which the reaction term is absent. The foundations of the theoretical model and calculation details are presented in [21], where subdiffusion of an antibiotic through a dense biofilm is described; in a dense biofilm antibiotic molecules can interact with bacteria throughout the biofilm volume. Here we present the model assumptions and basic equations. We use the function *W*_*B*_ describing the temporal evolution of the amount of a substance diffusing through the biofilm. This function takes different forms depending on whether or not antibiotic molecules are absorbed in the biofilm. To derive this function, the homogeneous biofilm approximation and the quasi-stationary approximation have been used [21]. Four stages in the process of transporting antibiotics through a biofilm have been defined by means of two criteria: (a) whether or not absorption of antibiotic particles in the biofilm occurs, (b) whether or not all biofilm parameters remain constant over time. The question we pose here is whether a model based on the above mentioned assumptions can be used to describe diffusion through a biofilm having a more heterogeneous structure. In fact, biofilms may be significantly inhomogeneous and can be thought of a ‘plumpudding geometry’, see below [22–24]. The modification of our mathematical model to describe antibiotic transport through a plumpudding–like biofilm is presented. In particular, we show that the sequence of stages for this biofilm is different than for a dense biofilm. Moreover, in one of the stages the function *W*_*B*_ for the dense biofilm and the ‘plumpudding biofilm’ are qualitatively different. Thus, the model is useful in determining the dynamics of antibiotic diffusion process through a biofilm and may even allow one to deduce the degree of heterogeneity of the biofilm under study.

The aim of the study is threefold.

(1) We show that the transport of an antibiotic in a biofilm can be described by a fractional subdiffusion-absorption equation. The ‘plumpudding biofilm’ is much more heterogeneous than a ‘dense biofilm’, but in the model we use the approximation of a homogeneous biofilm treating the transport parameters describing the process, the subdiffusion parameters *α* and *D*_*M*_ and the absorption coefficient *κ*, as non-local ‘effective parameters’ referring to the entire biofilm. We also use a quasistatic approximation, which in practice means that to describe the process we can use solutions of the subdiffusion-reaction equation with constant parameters, and then replace the parameters with time-dependent functions that are related to the changes in the biofilm structure. We show that the model gives theoretical results that are consistent with the experimental data.
(2) The approach here represent a non-invasive experimental method of studying the processes occurring in a biofilm, observing diffusion of an antibiotic in external regions outside the biofilm region. This method combines the ‘flow’ method described above with a theoretical model that predicts qualitatively different time evolution of the amount of antibiotic that has passed through the biofilm. As we mentioned before, the method shows whether or not absorption of the antibiotic in the biofilm occurs and whether or not the biofilm parameters change in time. Guided by these criteria we distinguish four stages of the process of antibiotic diffusion in the biofilm. Three of them will be observed in subdiffusion of ciprofloxacin through ASM with *Pseudomonas aeruginosa* biofilm structured as a plumpudding. We suppose that the transition from one stage to another is related to a qualitative change in the antibiotic-bacterial interaction. If the biofilm stops changing its state despite the continuous diffusion of the antibiotic into the biofilm, we conclude that the bacteria stopped responding to the antibiotic action. This scenario occurs in diffusion of ciprofloxacin through ASM with *Pseudomonas aeruginosa* biofilm. The substance concentration in this setup was measured by means of laser interferometry [25, 26].
(3) Using the method described in (2), we show that ciprofloxacin reacts with *Pseudomonas aeruginosa* bacteria found in the ‘plums’ located in the ASM. At a given initial concentration of the antibiotic, we determine the time after which the bacteria stop responding to the antibiotic action, which we interpret as the moment when the major portion of bacteria are incapacitated.

### A primer of biofilms

Biofilms are defined as complex microbial communities of cells embedded into a matrix of self-produced extracellular polymeric substance (EPS) with increased resistance to antibiotics and host immune response. This is an important issue because in many clinical and industrial settings biofilms represent a hazardous and costly problem. It is well documented that many chronic infections (∼65%), particularly those involving medical implants, causing urinary tract infections or in the course of cystic fibrosis involve biofilm-forming bacteria species [27–30]. Cystic fibrosis (CF) is a disease that causes thick, sticky mucus to build up in the lungs, the digestive tract, and other areas of the body. It is an optimal niche for microorganisms inducing chronic lung diseases in children and young adults, mainly by bacteria, such as *Pseudomonas aeruginosa* (80%), *Burkholderia cepacia* and *Staphylococcus aureus*. These bacterial infections lead to progressive pulmonary damage and emphysema. Eradication of bacterial biofilms formed in mucus is a crucial problem because the diffusion of classic antibiotics into biofilm structures is weak and their antibacterial activity might stimulate drug resistance, see Kindler et al. [31], Dilanji et al. [32], and Delle Side et al. [33] for additional references. Biophysical properties of biofilm structure EPS in gel-like mucus are directly associated with reduced susceptibility to antibiotics and limit the effective eradication of bacteria [34].

In this study, we focus on an experimental system mimicking physiological conditions typical to cystic fibrosis to measure antibiotic (ciprofloxacin) transport through a *P. aeruginosa* biofilm. Artificial sputum medium (ASM) is a culture medium mimicking CF patient sputum. This medium contains amino acids, mucin and free DNA. *P. aeruginosa* growth in this medium is very similar to sputum during CF infections. Moreover, natural interactions of sputum components with antibiotics used in bacteria eradication are observed in ASM, for example ciprofloxacin may bind to secretory mucin in sputum or epithelial mucin that lines airways, reducing free drug levels [35]. The most often used models of biofilms rely on surface attached biofilms whereas ASM biofilms resemble those observed in the CF lung. It seems to be crucial becuse the reduced oxygen concentration in the mucus has been shown to alter the behavior of *P. aeruginosa* and affect antibiotic susceptibility. PAO1 grown in ASM formed clusters or microcolonies, that are attached to the components of ASM but not the abiotic surface. This is thus an appropriate model of chronic lung colonization [23, 24].

## Materials and methods

**Fig 1:**
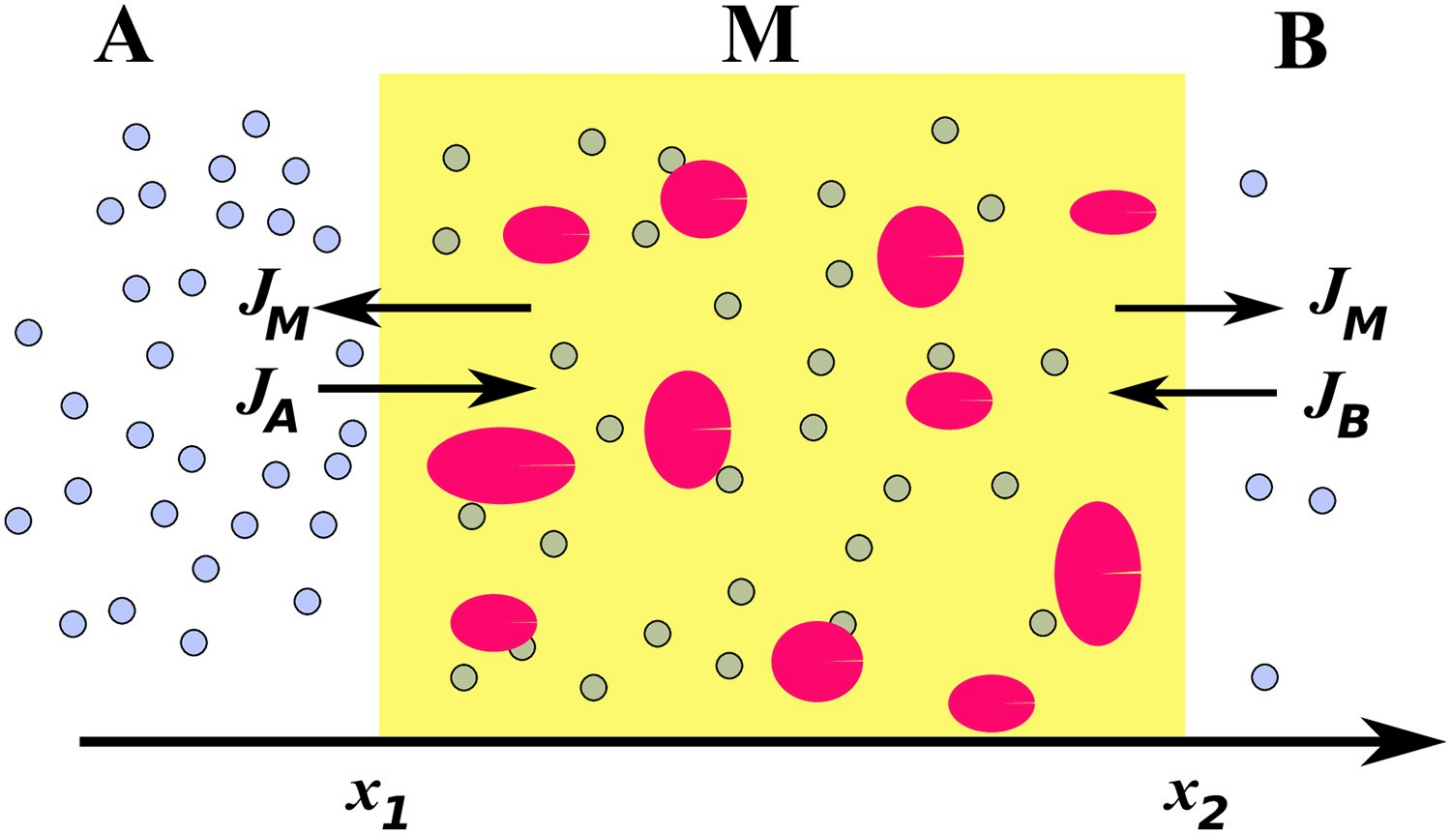
Diffusion of antibiotic molecules (blue circles) through a biofilm that looks like a plumpudding. The system consists of diffusive media *A* and *B* and the subdiffusive medium *M*, and *J* denotes the fluxes through the boundaries. The dark regions in *M* (plums) represent bacterial cells forming *Pseudomonas aeruginosa* biofilms which react with the antibiotic molecules. The pudding (light background) represents the artificial sputum medium which mimics the cystic fibrosis biofilm. The pudding is a diffusion barrier for antibiotic molecules in reaching the plums.

One of the main problems in the treatment of bacterial diseases is to check whether an antibiotic with a given initial concentration affects the bacteria, and if so, to determine the time after which the bacteria stop defending themselves against the effects of an antibiotic. It can be assumed that then the bacteria have been inactivated. In order to find the answer to this problem we study experimentally the time evolution of the amount of antibiotic *W*_*B*_ that has diffused from region *A* to *B* through the biofilm in a system presented schematically in Fig 1. The theoretical description is derived from a model based on the subdiffusion-absorption equation with fractional time derivative which describes antibiotic diffusion in a pure biofilm [21]. The theoretical model shows that the form of *W*_*B*_ depends on the presence of antibiotic molecule absorption in the biofilm. Absorption of antibiotic molecules and/or a change in biofilm parameters indicates that the bacteria sense the antibiotic and activate their defence mechanism against the antibiotic. Analysis of the function *W*_*B*_ obtained experimentally allows us to estimate the time after which the bacteria will react to the antibiotic action and the time when the bacteria cease to react.

### Experiment

**Fig 2:**
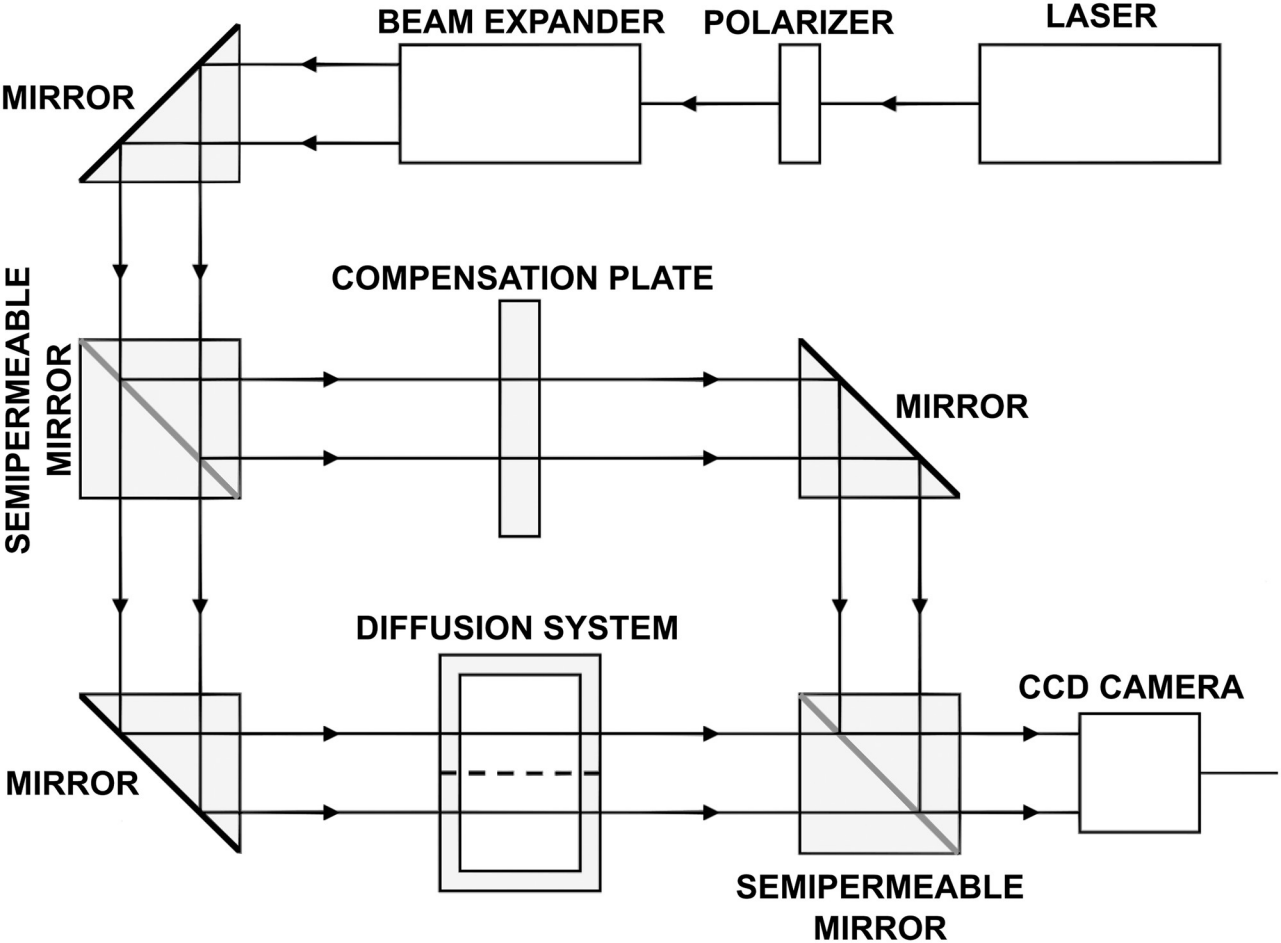
Scheme of the experimental setup, for its description see the text. The dashed horizontal line in the diffusion system represents the biofilm.

In Fig 2 a scheme of the experimental apparatus is presented, the ‘diffusion system’ corresponds to the system shown in Fig 1. The main element of the measurement set is the Mach-Zehnder interferometer. The light source is a helium-neon laser emitting light with wavelength λ = 628.8 nm. In order to adjust the light intensity to the sensitivity of the light detection system, the laser beam is weakened by the polarizer. Then it passes through a special expander, where it is transformed into a collimated coherent beam with a uniform distribution of intensity. Subsequently, the beam is separated by the input semipermeable mirror into two beams, one of which passes through the diffusion system under study and the other through the compensating plate. In one of the interferometer arms the tested diffusion system containing the solutions is placed. It consists of two glass cuvettes made from optical glass of very high homogeneity. In the other arm there is a glass plate that compensates the difference in optical paths brought in by the cuvette walls. Beams of light from both interferometer arms meet at the output semipermeable mirror, where they superimpose. The resulting interference images (interferograms) are registered by CCD camera and then analyzed by the computer system with special software. If in the tested diffusion system the solute is homogeneous (there are no concentration gradients), then as a result of beam interference we obtain an interference image in the form of a parallel straight-line fringes. The refractive index of the solution depends on the concentration of the diffusing substance. A concentration gradient generates a distribution of the refractive index in the solution and causes that the interference fringes are bent. The software examines the course of interference fringes, determines the deviation from their straight line run and calculates the values of the refractive index in different points in the solution. Using the relation between the substance concentration and the refractive index (determined by means of a refractometer in a separate experiment) the space-time distribution of concentration is determined [25, 26]. The measurement of the antibiotic concentration can be conducted with high precision in one of the regions *A* or *B* only. The reason is that the simultaneous measurement of the antibiotic concentration in *A* and *B* in the same experiment requires a very precise positioning of the measurement cuvettes in the axis of the laser beam. Then, the interference fringes in the upper and lower cuvettes are aligned with the same baseline. The slightest inaccuracy of the diffusion system alignment may have a negative impact on the measurement result. Therefore, we conduct the measure only in one region, here in region *B*.

We determine the temporal evolution of the function *W*_*B*_(*t*). This function was calculated numerically from experimental concentration profiles. The measurements of the concentration profiles were conducted in a vertically oriented vessel consisting of two glass cuboidlike cuvettes (70 mm high, 10 mm wide, 65 mm long) separated by a horizontally located PET membrane with PAO1 biofilm. The *P. aeruginosa* PAO1 mature biofilm was formed for 96 h at 37°C in artificial sputum medium (ASM) on the PET membrane with pore diameter 1 *μ*m, as an element of BD Falcon™ Cell Culture Inserts. ASM was formulated to mimic the sputum of cystic fibrosis (CF) patients and to study microbial biofilms, for example, *Pseudomonas aeruginosa* colonization in CF lungs [36]. At the first step of the analysis, the percentage of the membrane covered by PAO1 biofilm was estimated. The images of the membrane covered by PAO1 biofilm were stained by CV (0.004%) for 15 min, converted to grey-scale digital images and analyzed with the ImageJ computer imaging software program [37]. Initially, the upper cuvette was filled with an aqueous solution of ciprofloxacin with *C*_0_ = 1 mg/ml = 3.02 mol/m^3^, whereas the lower cuvette was filled with pure water. Since the concentration gradient is solely in the vertical direction, antibiotic transport is effectively one dimensional.

### Theory

We consider antibiotic diffusion in the system shown in Fig 1. We assume that the system is homogeneous in a plane perpendicular to the *x* axis, thus the problem is effectively one–dimensional. The system consists of the three regions *A* (*x* < *x*_1_), *M* (*x*_1_ < *x* < *x*_2_), and *B* (*x* > *x*_2_); these symbols also label the concentrations in the relevant parts of the system. At the initial moment part *A* contains a homogeneous aqueous solution of antibiotic (ciprofloxacin), while part *B* is filled with pure water, the region *M* is free of antibiotic. The initial concentrations are thus

$$\begin{array}{c} C_{A}\left(x,0\right)=C_{0},\;C_{M}\left(x,0\right)=C_{B}\left(x,0\right)=0. \end{array}$$ (1)

The equations which describe the process are the normal diffusion equation in regions *A* and *B*

$$\begin{array}{c} \frac{\partial C_{A,B}\left(x,t\right)}{\partial t}=D\frac{\partial^{2}C_{A,B}\left(x,t\right)}{\partial x^{2}} \end{array}$$ (2)

with diffusion coefficient *D*, and the time–fractional diffusion–absorption equation

$$\begin{array}{c} \frac{\partial C_{M}\left(x,t\right)}{\partial t}=D_{M}\frac{\partial^{1-\alpha }}{\partial t^{1-\alpha }}[\frac{\partial^{2}C_{M}\left(x,t\right)}{\partial x^{2}}-\kappa^{2}C_{M}\left(x,t\right)], \end{array}$$ (3)

where *α*, *D*_*M*_, and *κ* denote the subdiffusion parameter, the subdiffusion coefficient, and the absorption coefficient, respectively, in the medium *M*. *D*_*M*_ and *κ* have physical dimension [*D_M_*] = length^2^/time*α* and [*κ*] = 1/length, respectively. Eq (3) contains the Riemann–Liouville fractional time derivative of order 1 − *α* [38]. The Riemann–Liouville fractional derivative is defined for 0 < *β* < 1 as

$$\frac{d^{\beta }f\left(t\right)}{dt^{\beta }}=\frac{1}{\Gamma (1-\beta )}\frac{d}{dt}\int_{0}^{t}dt^{\prime }\frac{f(t^{\prime })}{{\left(t-t^{\prime }\right)}^{\beta }}\;.$$

We mention that for *κ* = 0 and in an infinite medium Eq (3) encodes the mean square displacement 〈(Δ*x*)^2^〉 = 2*D*_*M*_ *t*^*α*^/Γ(1 + *α*) such that for 0 < *α* < 1 we have subdiffusion and for *α* = 1 there is normal diffusion. The appearance of the fractional time derivative in the subdiffusion equation means that the process has a long memory [38]. In a random walk picture the particle is hindered in a subdiffusion medium, the sojourn time distribution for the next jump of the particle *ψ* has a heavy tail, *ψ*(*t*)∼1/*t*^1+ *α*^ when *t* → ∞. Then, the mean value of this time is infinite [38].

To describe diffusion-absorption in a biofilm, we use the approximation of a homogeneous medium *M*. The parameters *α*, *D*_*M*_, and *κ* are effective parameters describing diffusion and absorption throughout the medium; they represent antibiotic diffusion through the matrix and the interaction with the biofilm plums. The boundary conditions at the border between the media, which were derived in [21], are

$$\begin{array}{c} \left(1-q_{A}\right)DC_{A}\left(x_{1}^{-},t\right)=D_{M}\frac{\partial^{1-\alpha }C_{M}\left(x_{1}^{+},t\right)}{\partial t^{1-\alpha }}, \end{array}$$ (4)

$$\begin{array}{c} J_{A}\left(x_{1}^{-},t\right)=J_{M}\left(x_{1}^{+},t\right), \end{array}$$ (5)

$$\begin{array}{c} D_{M}\frac{\partial^{1-\alpha }C_{M}\left(x_{2}^{-},t\right)}{\partial t^{1-\alpha }}=\left(1-q_{B}\right)DC_{B}\left(x_{2}^{+},t\right), \end{array}$$ (6)

$$\begin{array}{c} J_{M}\left(x_{2}^{-},t\right)=J_{B}\left(x_{2}^{+},t\right), \end{array}$$ (7)

where the diffusive fluxes are defined as *J*_*A*,*B*_(*x*, *t*) = −*D*∂*C*_*A*,*B*_(*x*, *t*)/∂*x* and *J*_*M*_(*x*, *t*) = −*D*_*M*_(∂^1−*α*^/∂*t*^1−*α*^)∂*C*_*M*_(*x*, *t*)/∂*x*. The boundary conditions mean that the diffusion flux at the biofilm surface is continuous and that the antibiotic molecule that tries to leave the biofilm can do so without any obstacles, but its passage into the biofilm surface in the opposite direction can be made with asymmetric probabilities 1 − *q*_*A*_ (for the biofilm surface located at *x*_1_) and 1 − *q*_*B*_ (at *x*_2_) [21, 39].

In the experiment, we determine the temporal evolution of the function $W_{B}\left(t\right)=\Pi \int_{x_{2}}^{\infty }C_{B}\left(x,t\right)dx$, where Π is the biofilm surface area. In [21] it was shown that the form of the function *W*_*B*_ depends on whether or not absorption takes place in the medium *M*. Four stages of the diffusion process of the antibiotic through the biofilm were distinguished, taking into account the following ‘physical’ criteria: (1) whether or not absorption of antibiotic molecules in the biofilm is observed, and (2) if the biofilm parameters are constant or if at least one of them changes over time. Since the function *W*_*B*_ is qualitatively different at each stage, it serves to identify these stages in the process under consideration. For a sufficiently long time, this function takes the following forms when the biofilm parameters are constant [21]

$$\begin{array}{cc} W_{0B}\left(t\right)=C_{0}\Pi (a_{0}\sqrt{t}-b_{0}t^{1-\alpha }), & \kappa =0\;, \end{array}$$ (8)

$$\begin{array}{cc} W_{\kappa B}\left(t\right)=C_{0}\Pi (a_{\kappa }-\frac{b_{\kappa }}{\sqrt{t}}-\frac{c_{\kappa }}{t^{\alpha }}), & \kappa \neq 0\;, \end{array}$$ (9)

where $a_{0}=2\left(1-{\tilde{q}}_{A}\right)\sqrt{D}/\left(2-{\tilde{q}}_{A}-{\tilde{q}}_{B}\right)\sqrt{\pi }$, $b_{0}=a_{0}^{2}\pi d\left(1-{\tilde{q}}_{B}\right)/2{\tilde{D}}_{M}\Gamma \left(2-\alpha \right)$, *a_κ_* = 1/(1 − *q_B_*)*κ* sinh(*κd*), $b_{\kappa }=a_{\kappa }\coth\left(\kappa d\right)\left[1/\left(1-q_{A}\right)+1/\left(1-q_{B}\right)\right]/\sqrt{\pi D}$, *c*_*κ*_ = *a*_*κ*_[1+ *κd*coth(*κd*)]/2*κ*^2^ *D*_*M*_ Γ(1 − *α*), and *d* = *x*_2_ − *x*_1_. In the above equations, we took into account that the biofilm coefficients may be different for the cases described by Eqs (8) and (9). When *κ* = 0, the coefficients are denoted as ${\tilde{D}}_{M}$, ${\tilde{q}}_{A}$, and ${\tilde{q}}_{B}$.

When absorption occurs and the biofilm parameters change over time, the following function has been proposed [21]$W_{\tilde{\kappa }\left(t\right)B}\left(t\right)=\rho \left(t\right)W_{\kappa B}\left(t\right)$, where *ρ*(*t*) is to be determined from empirical data. For the case considered in this paper, the function *ρ*(*t*) = *a* − *b*/*t* gives a good agreement of the *W*_*B*_ function and experimental results in the time interval 〈*t*_1_, *t*_2_〉. The times *t*_1_ and *t*_2_ separating the subsequent stages are defined by the equations *W*_0*B*_(*t*_1_) = *W*_*κB*_(*t*_1_) and $W_{\kappa B}\left(t_{2}\right)=W_{\tilde{\kappa }\left(t\right)B}\left(t_{2}\right)$. Thus, when *κ* ≠ *const*. ≠ 0, *W*_*B*_ takes the form

$$\begin{array}{c} W_{\tilde{\kappa }\left(t\right)B}\left(t\right)=C_{0}\Pi (a-\frac{b}{t})(a_{\kappa }-\frac{b_{\kappa }}{\sqrt{t}}-\frac{c_{\kappa }}{t^{\alpha }})\;. \end{array}$$ (10)

The parameters *a*_*κ*_, *b*_*κ*_, and *c*_*κ*_ for the functions Eqs (9) and (10) are taken to be identical. We also assume that the parameter *α* in Eqs (8), (9) and (10) is the same. The stage without absorption and changing biofilm parameters has not been observed here, such that our observations split into three stages.

We suppose that the absorption probability of antibiotic molecules per unit length of biofilm, which is controlled by the parameter *κ*, and the biofilm thickness *d* are small, *κd* ≪ 1 [21]. If the parameters *D* and *α* are constant, by swapping the other parameters in Eq (9) according to the formula

$$\begin{array}{c} \kappa \to \frac{\kappa }{\rho (t)},\;1-q_{A,B}\to \left(1-q_{A,B}\right)\rho \left(t\right),\;D_{M}\to D_{M}\rho^{2}\left(t\right), \end{array}$$ (11)

we obtain Eq (10).

## Results and discussion

**Fig 3:**
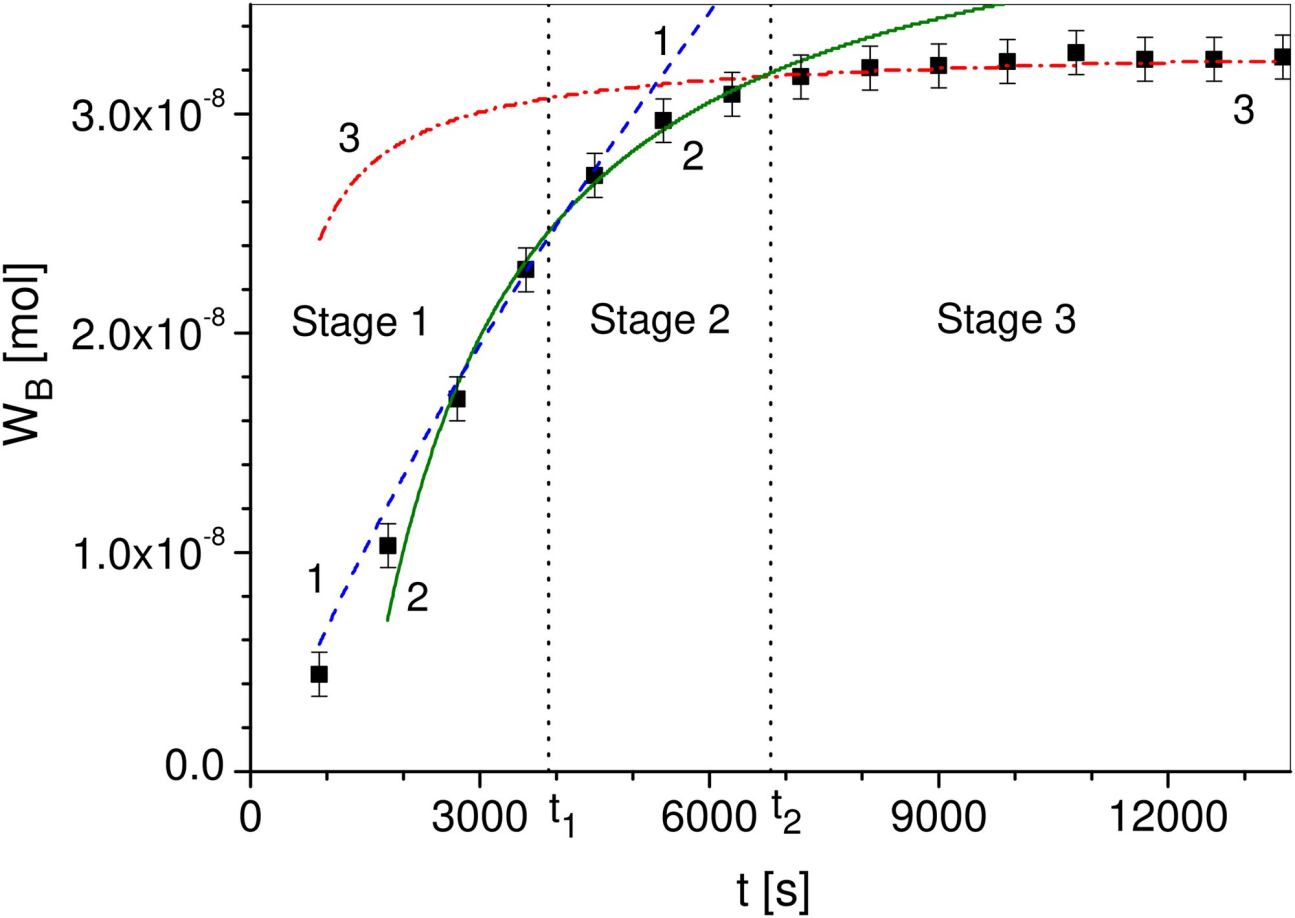
The amount of ciprofloxacin *W*_*B*_ which diffuses into part *B*. Points represent the experimental data (see S1 File) and lines represent theoretical functions. Line No. 1 (dashed line) corresponds to function *W*_0*B*_ Eq (8) for $a_{0}=2.79\times 10^{-6}\;m/\sqrt{s}$ and *b*_0_ = 4.21 × 10^−5^ m/s^0.04^, line No. 2 (solid line) represents $W_{\tilde{\kappa }\left(t\right)B}$ Eq (10) for *a_κ_* = 1.59 × 10^−4^ m, $b_{\kappa }=3.53\times 10^{-4}\;m\sqrt{s}$ and *c*_κ_ = 2.22 × 10^−2^ m s^0.96^, *a* = 1.28, and *b* = 1850 s, line No. 3 (dashed–dotted line) represents *W*_*κB*_ Eq (9), *t*_1_ = 3900 s, *t*_1_ = 6800 s, *C*_0_ = 3.02 mol/m^3^, and Π = 7.0 × 10^−5^ m^2^. For all cases *α* = 0.96. The two vertical dotted lines are the boundaries between the stages.

As already mentioned, the setup of our experiment following Figs 1 and 2 is fully non-invasive, and our central observable is *W*_*B*_, the amount of substance that has diffused into part *B*. The experimental results are shown in Fig 3. Points represent the experimental data, which have been calculated from the experimentally measured antibiotic concentration profiles, and lines represents the theoretical functions Eqs (8)–(10). We fit the theoretical functions to the experimental data and find the values of the parameters given in the figure caption. In all cases, the subdiffusion coefficient *α* = 0.96 is the same. This parameter has been determined as one of the fitting parameters when analyzing Stage 1. Unfortunately, the statistic is too poor to determine the error for *α*. Because the consistency of ASM is very similar to the consistency of 1% aqueous agarose solution for which *α* = 0.95 [40], we suppose that there is subdiffusion in the ASM medium and the value of the parameter *α* is realistic. We note that anomalous diffusion exponents as about of 0.9 are not exceptional and physically meaningful, see e.g. [14, 41, 42]. The remaining parameters have been determined by fitting the theoretical functions to the experimental results as follows. Parameters *a*_0_ and *b*_0_ provide the best fit of the function *W*_0*B*_ Eq (8), line No. 1 in Fig 3, to empirical data. Similarly, parameters *a*_*κ*_, *b*_*κ*_, and *c*_*κ*_ provide the best fit of the function *W*_*κB*_ Eq (9), line No. 2 in Fig 3, the same parameters have been used in the function $W_{\tilde{\kappa }\left(t\right)B}$ Eq (10), line No. 3, but the fit parameters are *a* and *b* here.

The observed order of the stages in Fig 3 is as follows: initially we see *Stage 1* described by Eq (8), followed by *Stage 2* described by Eq (10), and finally we see *Stage 3* described by Eq (9). We note that the function *ρ*(*t*) is increasing over time. Due to Eq (11), the parameter *κ* is a function decreasing over time and the parameter *D*_*M*_ increases over time in Stage 2. The interpretation of the process is that at Stage 1 there is a sub-inhibitory concentration of the antibiotic near the bacteria located in the biofilm plums. The bacterial defence mechanisms are then not fully activated. We mention that *W*_0*B*_ in Eq (8) also describes antibiotic diffusion through ASM in which bacteria would not be present. The initial conditions Eq (1) cause a continuous diffusion of the antibiotic from the region *A* to the regions *M* and *B*. We therefore assume that the concentration of the antibiotic in ASM increases with time. Thus, in Stage 2 the defence mechanisms is activated due to the increasing of antibiotic concentration near the plums. The antibiotic effect weakens the bacteria to such a level that the influence of factors causing absorption of antibiotic molecules and slowing down of diffusion decreases, which causes changes in the biofilm parameters. At the moment *t*_2_ these parameters stop changing, which can be interpreted as the inability of bacteria to continue their active defence.

## Conclusion

We have shown a method to experimentally check whether a biofilm in a plumpudding scenario absorbs antibiotic particles and whether the biofilm parameters change over time. The presented three-stage model describes subdiffusion of ciprofloxacin through ASM with *Pseudomonas aeruginosa* bacteria. However, when studying other processes it may be possible to observe the fourth stage in which there is no absorption and the parameters of the biofilm change over time; this stage is not observed in our date in Fig 3. The division into stages is based on the physical aspects of the process. A biological interpretation of the processes described by physical models seems to be a difficult task. It is not obvious which bacterial defence mechanisms can be included in these stages, see [6, 7, 43–45]. However, we believe that determining the order of the stages may facilitate the biological interpretation of the process for various antibiotic-biofilm systems. We mention a few hypotheses in which the bacterial defence mechanisms can occur in different stages. When an antibiotic molecule enters the bacteria and then is excreted through them, as it happens in the efflux pump mechanism, it causes some delay in the random walk of the molecule resulting in molecule diffusion with a constant diffusion coefficient, absorption process does not occur there. If absorption does not occur but the subdiffusion parameters change in time, the reaction of the bacteria can be a gradual thickening of the biofilm. The formation of a diffusion barrier is one of the defence mechanisms of bacteria [6, 7, 43, 45, 46]. When the biofilm density becomes very high, it is possible to permanently retain antibiotic molecules in the biofilm, which is interpreted as absorption of the molecule. There are many complex bacterial defence mechanisms in the biofilm [6–8, 31, 47, 48], their assignment to individual stages of the process requires additional consideration. However, we also believe that the presented experimental method may be useful when planning treatment for cystic fibrosis patients who also have another bacterial infection. In particular, this method allows to determine the time at which the bacteria intensify the process of defence against the antibiotic and the time at which this process is stopped, these times certainly depend on the antibiotic concentration.

A different course of the process has been observed when a ‘dense biofilm’ attacked by bacteria fills the entire space of the central part *M* of the system. In [21] it is shown that in this case the order of the stages is as follows (we keep the notation used in the present paper): Stage 1, Stage 3, then Stage 2. The function *ρ*(*t*) is decreasing, so the absorption coefficient increases while the subdiffusion coefficient decreases over time. The reason that the course of antibiotic diffusion in a dense biofim and in the plumpudding scenario is qualitatively different is as follows. In a dense biofilm, antibiotic molecules can ‘feel the action’ of the bacterial defence mechanisms the whole time inside the biofilm. In a plumpudding case, an antibiotic molecule feels the defence mechanism after reaching a plum occupied by bacteria. This plum can be attacked intensively, from all sides, by an antibiotic which concentration at the surface may increase over time due to diffusion process. This leads to a weakening of the bacterial defences faster than in a dense biofilm. We hypothesize that the order of states, which may be checked experimentally, is indicative of the structure of the biofilm, namely whether or not it can be treated as a dense biofilm. However, since there are a lot of mechanisms for the defence of bacteria against the effects of an antibiotic, whether the order of stages in both cases is universal should receive further study along with improved experiments.

## Supporting information

S1 File (PDF)

## References

[1] Kolesov G, Wunderlich Z, Laikova ON, Gelfand MS, Mirny LA. How gene order is influenced by the biophysics of transcription regulation. PNAS. 2007 8;104(35):13948–13953. www.pnas.orgcgidoi10.1073pnas.0700672104. 17709750 10.1073/pnas.0700672104 PMC1955771

[2] Pulkkinen O, Metzler R. Distance matters: the impact of gene proximity in bacterial gene regulation. Physical Review Letters 2013 5;110(19):198101 10.1103/PhysRevLett.110.198101 23705743

[3] Holcman D, Schuss Z. 100 years after Smoluchowski: stochastic processes in cell biology. Journal of Physics A 2017 1;50:093002 10.1088/1751-8121/50/9/093002

[4] Grebenkov DS, Metzler R, Oshanin G. Strong defocusing of molecular reaction times results from an interplay of geometry and reaction control. Communications Chemist ry 2018 12;1:96 10.1038/s42004-018-0096-x—www.nature.com/commschem.

[5] Suzuki T. Mathematical Methods for Cancer Evolution. Springer Nature Singapore.; 2017.

[6] Anderson GG, O’Toole GA. Innate and induced resistance mechanisms of bacterial biofilms. Current Topics in Microbiology and Immunology. 2008 322:85–105. http://link.springer.com/10.1007/978-3-540-75418-3 18453273 10.1007/978-3-540-75418-3_5

[7] Mah TFC, O’Toole GA. Mechanisms of biofilm resistance to antimicrobial agents. Trends in Microbiology. 2001 1;9(1):34–39. 10.1016/S0966-842X(00)01913-2 11166241

[8] Stewart PS, White B, Boegli L, Hamerly T, Williamson KS, Franklin MJ et al Conceptual model of biofilm antibiotic tolerance that integrates phenomena of diffusion, metabolism, gene expression, and physiology. Journal of Bacteriology. 2019 11;201(22):e00307–19. 10.1128/JB.00307-19 31501280 PMC6805107

[9] Balouiri M, Sadiki M, Ibnsouda SK. Methods for in vitro evaluating antimicrobial activity: A review. Journal of Pharmaceutical Analysis. 2016 4;6(2):71–79. 10.1016/j.jpha.2015.11.005 29403965 PMC5762448

[10] Purevdorj B, Costerton JW, Stoodley P. Influence of hydrodynamics and cell signaling on the structure and behavior of Pseudomonas aeruginosa biofilms. Applied and Environmental Microbiology. 2002 9;68:4457–4464. 10.1128/AEM.68.9.4457-4464.2002 12200300 PMC124093

[11] Olszak T, Danis-Wlodarczyk K, Arabski M, Gula G, Maciejewska B, Wasik S, et al Pseudomonas aeruginosa PA5oct jumbo phage impacts planktonic and biofilm population and reduces its host virulence. Viruses 2019 11;11(12):1089 10.3390/v11121089. 31771160 PMC6950013

[12] Olszak T, Shneider MM, Latka A, Maciejewska B, Browning C, Sycheva LV, et al The O-specific polysaccharide lyase from the phage LKA1 tailspike reduces Pseudomonas virulence. Scientific Reports. 2017 11;24;7(1):16302. 10.1038/s41598-017-16411-4 PMC5701251 29176754

[13] Danis-Wlodarczyk K, Olszak T, Arabski M, Wasik S, Majkowska-Skrobek G, Augustyniak D, et al Characterization of the newly isolated lytic bacteriophages KTN6 and KT28 and their efficacy against Pseudomonas aeruginosa biofilm. PLoS One. 2015 5;21;10(5):e0127603 10.1371/journal.pone.0127603 25996839 PMC4440721

[14] Kosztołowicz T, Dworecki K, Mrówczyński S. How to measure subdiffusion parameters. Physical Review Letters. 2005 5;94:170602 10.1103/PhysRevLett.94.170602 15904275

[15] Alcazar-Cano N, Delgado-Buscalioni R. A general phenomenological relation for the subdiffusive exponent of anomalous diffusion in disordered media. Soft Matter 2018 11;14(48):9937–9949. 10.1039/C8SM01961D 30488923

[16] Lieleg O, Vladescu I, Ribbeck K. Characterization of particle translocation through mucin hydrogels. Biophysical Journal. 2010 5;98(9):1782–1789. 10.1016/j.bpj.2010.01.012 20441741 PMC2862156

[17] Wong IY, Gardel ML, Reichman DR, Weeks ER, Valentine MT, Bausch AR, et al Anomalous diffusion probes microstructure dynamics of entangled F-actin networks. Physical Review Letters. 2004 4; 92(17):178101 10.1103/PhysRevLett.92.178101 15169197

[18] Godec A, Bauer M, Metzler R. Collective dynamics effect transient subdiffusion of inert tracers in flexible gel networks. New Journal of Physics. 2014 9;16:092002 10.1088/1367-2630/16/9/092002

[19] Jeon JH, Leijnse N, Oddershede LB, Metzler R. Anomalous diffusion and power-law relaxation of the time averaged mean squared displacement in worm-like micellar solutions. New Journal of Physics. 2013 4;15:045011 10.1088/1367-2630/15/4/045011.

[20] Cherstvy AG, Thapa S, Wagner CE, Metzler R. Non-Gaussian, non-ergodic, and non-Fickian diffusion of tracers in mucin hydrogels. Soft Matter 2019 3;15:2526–2551. 10.1039/C8SM02096E. 30734041

[21] Kosztołowicz T, Metzler R. Diffusion of antibiotics through a biofilm in the presence of diffusion and absorption barriers. Physical Review E 2020 9;102:032408 10.1103/PhysRevE.102.032408 33075880

[22] Stewart PS, Franklin MJ. Physiological heterogeneity in biofilms. Nature Reviews Microbiology 2008 3;6(3):199–210. 10.1038/nrmicro1838 18264116

[23] Sriramulu DD, Lűnsdorf H, Lam JS, Römling U. Microcolony formation: a novel biofilm model of Pseudomonas aeruginosa for the cystic fibrosis lung. Journal of Medical Microbiology. 2005 7;54(7):667–676. 10.1099/jmm.0.45969-0 15947432

[24] Haley CL, Colmer-Hamood JA, Hamood AN. Characterization of biofilm-like structures formed by Pseudomonas aeruginosa in a synthetic mucus medium. BMC Microbiology. 2012 8;12:181 10.1186/1471-2180-12-181 22900764 PMC3494610

[25] Arabski M, Wa̡sik S, Dworecki K, Kaca W. Laser interferometric determination of ampicillin and colistin transfer through cellulose biomembrane in the presence of Proteus vulgaris O25 lipopolysaccharide. Journal of Membrane Sciences 2007 8;299(1-2):268–275. 10.1016/j.memsci.2007.05.003.

[26] Wa̡sik S, Arabski M, Drulis-Kawa Z, Gubernator J. Laser interferometry analysis of ciprofloxacin and ampicillin diffusion from liposomal solutions to water phase. European Biophysics Journal 2013 4;42:549–558. 10.1007/s00249-013-0904-2 23604440 PMC3674336

[27] Lesouhaitier O, et al Host peptidic hormones affecting bacterial biofilm formation and virulence. Journal of Innate Immunity 2019 11;11:227–241. 10.1159/000493926 30396172 PMC6738206

[28] Parsek MR, Tolker-Nielsen T. Pattern formation in Pseudomonas aeruginosa biofilms. Current Opinion in Microbiology 2008 11;11(6):560–566. 10.1016/j.mib.2008.09.015 18935979

[29] Sauer K, Camper AK, Ehrlich GD, Costerton JW, Davies DG. Pseudomonas aeruginosa displays multiple phenotypes during development as a biofilm. Journal of Bacteriology. 2002 2;184(4):1140–1154. 10.1128/jb.184.4.1140-1154.2002 11807075 PMC134825

[30] Costerton JW. Cystic fibrosis pathogenesis and the role of biofilms in persistent infection. Trends in Microbiology. 2001 2;9(2):50–52. 10.1016/S0966-842X(00)01918-1 11173226

[31] Kindler O, Pulkkinen O, Cherstvy AG, Metzler R. Burst statistics in an early biofilm quorum sensing model: the role of spatial colony-growth heterogeneity. Scientific Reports. 2019 8;9:12077 10.1038/s41598-019-48525-2 31427659 PMC6700081

[32] Dilanji GE, Langebrake JB, De Leenheer P, Hagen SJ. Quorum activation at a distance: spatiotemporal patterns of gene regulation from diffusion of an autoinducer signal. Journal of the American Chemical Society 2012 3;134(12):5618–5626. 10.1021/ja211593q 22372494

[33] Delle Side D, Nassisi V, Pennetta C, Alifano P, Di Salvo M, Tala A, et al Bacterial bioluminescence onset and quenching: a dynamical model for a quorum sensing-mediated property. Royal Society Open Science 2017 12;4:171586 10.1098/rsos.171586. 29308273 PMC5750040

[34] Stewart PS. Diffusion in biofilms. Journal of Bacteriology. 2003 3;185(5):1485–1491. 10.1128/JB.185.5.1485-1491.2003 12591863 PMC148055

[35] Huang JX, Blaskovich MA, Pelingon R, et al Mucin binding reduces colistin antimicrobial activity. Antimicrobial Agents and Chemotherapy. 2015 9;59(10):5925–5931. 10.1128/AAC.00808-15 26169405 PMC4576126

[36] Kirchner S, Fothergill JL, Wright EA, James CE, Mowat E, Winstanley C. Use of artificial sputum medium to test antibiotic efficacy against Pseudomonas aeruginosa in conditions more relevant to the cystic fibrosis lung. Journal of Visualized Experiments. 2012 6;64:e3857. 10.3791/3857 PMC3471314 22711026

[37] Schneider CA, Rasband WS, Eliceiri KW. NIH Image to ImageJ: 25 years of image analysis. Nature Methods. 2012 6;9:671675. 10.1038/nmeth.2089 PMC5554542 22930834

[38] Metzler R, Klafter J. The random walk’s guide to anomalous diffusion: a fractional dynamics approach. Physics Reports. 2000 12;339:1–77. 10.1016/S0370-1573(00)00070-3

[39] Kosztołowicz T. Model of anomalous diffusion-absorption process in a system consisting of two different media separated by a thin membrane. Physical Review E. 2019 2;99:022127 10.1103/PhysRevE.99.022127 30934262

[40] Kosztołowicz T. Subdiffusion in a system consisting of two different media separated by a thin membrane. International Journal of Heat and Mass Transfer. 2017 8;111:1322–1333. 10.1016/j.ijheatmasstransfer.2017.04.058.

[41] Weigel AV, Simon B, Tamkun MM, Krapf D. Ergodic and nonergodic processes coexist in the plasma membrane as observed by single-molecule tracking. Proceedings of the National Academy of Sciences. 2011 4;108(16):6438–6443. 10.1073/pnas.1016325108. 21464280 PMC3081000

[42] Jeon JH, Martinez-Seara Monne H, Javanainen M, Metzler R. In vivo anomalous diffusion and weak ergodicity breaking of lipid granules. Physical Review Letters. 2012 10;109:188103 10.1103/PhysRevLett.106.048103. 21405366

[43] Chambless JD, Hunt SM, Stewart PS. A three-dimensional computer model of four hypothetical mechanisms protecting biofilms from antimicrobials. Applied and Environmental Microbiology. 2006 3;72(3):2005–2013. 10.1128/AEM.72.3.2005-2013.2006 16517649 PMC1393201

[44] Stewart PS. Biofilm accumulation model that predicts antibiotic resistance of Pseudomonas aeruginosa biofilms. Antimicrobial Agents and Chemotherapy. 1994 5;38(5):1052–1058. 10.1128/AAC.38.5.1052 8067737 PMC188149

[45] Stewart PS. Theoretical aspects of antibiotic diffusion into microbial biofilms. Antimicrobial Agents and Chemotherapy. 1996 11;40(11):2517–2522 10.1128/AAC.40.11.2517 8913456 PMC163567

[46] Jacobs M, Gregoire N, Couet W, Bulitta JB. Distinguishing antimicrobial models with different resistance mechanisms via population pharmacodynamic modeling. PLoS Computational Biology. 2016 3;12(3):e1004782 10.1371/journal.pcbi.1004782. 26967893 PMC4788427

[47] Lu S, Liu F, Xing B, Yeow EKL. Nontoxic colloidal particles impede antibiotic resistance of swarming bacteria by disrupting collective motion and speed. Physical Review E. 2015 12;92:062706 10.1103/PhysRevE.92.062706. 26764726

[48] Singla S, Harjai K, Chhibber S. Susceptibility of different phases of biofilm of Klebsiella pneumoniae to three different antibiotics. Journal of Antibiotics. 2013 2;66(2):61–66. 10.1038/ja.2012.101 23168403

