# Supplementary material for: Modelling experimentally measured of ciprofloxacin antibiotic diffusion in Pseudomonas aeruginosa biofilm formed in artificial sputum medium
Source: PLoS One. 2020 Dec 3;15(12):e0243003. doi: 10.1371/journal.pone.0243003 (PMC7714214; doi:10.1371/journal.pone.0243003)
Supplement: S1 File — (PDF) [file pone.0243003.s001.pdf]

Diffusion of ciprofloxacin through ASM

t [sec]	$W_B(t)$ [mol]
900	4.44E-09
1800	1.03E-08
2700	1.70E-08
3600	2.29E-08
4500	2.72E-08
5400	2.97E-08
6300	3.09E-08
7200	3.17E-08
8100	3.21E-08
9000	3.22E-08
9900	3.24E-08
10800	3.28E-08
11700	3.25E-08
12600	3.25E-08
13500	3.26E-08
